# {6,6′-Dieth­oxy-2,2′-[4,5-dimethyl-*o*-phenyl­enebis(nitrilo­methyl­idyne)]diphenolato}copper(II)

**DOI:** 10.1107/S1600536810042789

**Published:** 2010-10-30

**Authors:** Arezoo Jamshidvand, Hadi Kargar, Reza Kia, Muhammad Nawaz Tahir

**Affiliations:** a Department of Chemistry, School of Science, Payame Noor University (PNU), Ardakan, Yazd, Iran; b Department of Chemistry, Science and Research Branch, Islamic Azad University, Tehran, Iran; c X-ray Crystallography Lab., Plasma Physics Research Center, Science and Research Branch, Islamic Azad University, Tehran, Iran; d Department of Physics, University of Sargodha, Punjab, Pakistan

## Abstract

In the title complex, [Cu(C_26_H_26_N_2_O_4_)], the Cu^II^ ion lies on a crystallographic twofold rotation axis and is coordinated in a slightly distorted square-planar environment. The dihedral angle between the central benzene ring and each of the two symmetry-related outer benzene rings is 5.1 (2)°. The crystal structure is stabilized by inter­molecular π–π inter­actions with centroid–centroid distances in the range 3.466 (2)–3.6431 (16) Å.

## Related literature

For background to Schiff base–metal complexes, see: Granovski *et al.* (1993 ▶); Blower *et al.* (1998 ▶); Elmali *et al.* (2000 ▶). For standard bond lengths, see: Allen *et al.* (1987 ▶).
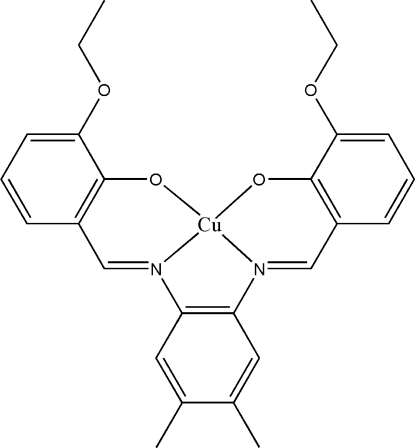


## Experimental

### 

#### Crystal data

[Cu(C_26_H_26_N_2_O_4_)]
*M* *_r_* = 494.03
Monoclinic, 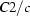

*a* = 14.9755 (7) Å
*b* = 15.8803 (7) Å
*c* = 12.2264 (6) Å
β = 119.285 (2)°
*V* = 2536.0 (2) Å^3^
*Z* = 4
Mo *K*α radiation
μ = 0.89 mm^−1^
*T* = 296 K
0.27 × 0.21 × 0.11 mm

#### Data collection

Bruker SMART APEXII CCD area-detector diffractometer
Absorption correction: multi-scan (*SADABS*; Bruker, 2005 ▶) *T*_min_ = 0.982, *T*_max_ = 0.992
31403 measured reflections
3157 independent reflections
1910 reflections with *I* > 2σ(*I*)
*R*_int_ = 0.049

#### Refinement

*R*[*F* ^2^ > 2σ(*F* ^2^)] = 0.066
*wR*(*F* ^2^) = 0.147
*S* = 1.05
3157 reflections
152 parameters
H-atom parameters constrained
Δρ_max_ = 0.43 e Å^−3^
Δρ_min_ = −0.46 e Å^−3^

### 

Data collection: *APEX2* (Bruker, 2005 ▶); cell refinement: *SAINT* (Bruker, 2005 ▶); data reduction: *SAINT*; program(s) used to solve structure: *SHELXTL* (Sheldrick, 2008 ▶); program(s) used to refine structure: *SHELXTL*; molecular graphics: *SHELXTL*; software used to prepare material for publication: *SHELXTL* and *PLATON* (Spek, 2009 ▶).

## Supplementary Material

Crystal structure: contains datablocks global, I. DOI: 10.1107/S1600536810042789/lh5150sup1.cif

Structure factors: contains datablocks I. DOI: 10.1107/S1600536810042789/lh5150Isup2.hkl

Additional supplementary materials:  crystallographic information; 3D view; checkCIF report

## Figures and Tables

**Fig. 1.**
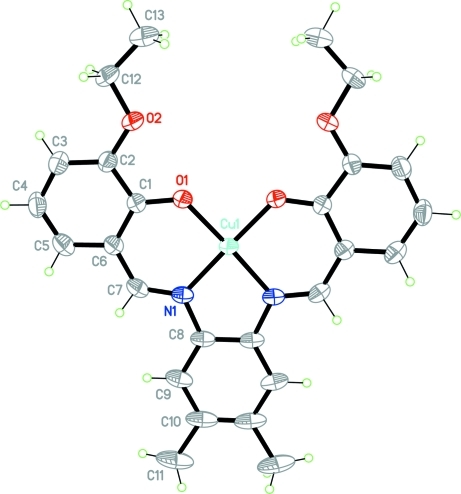
The molecular structure of the title compound, showing 40% probability displacement ellipsoids and the atomic numbering. Symmetry code for the unlabled atoms: -x, y, -z + 1/2

**Fig. 2.**
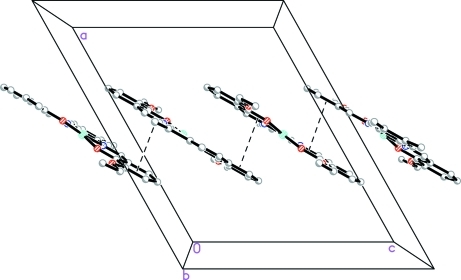
Part of the crystal structure viewed approximately along the b-axis showing π–π stacking interactions as dashed lines.

## supplementary crystallographic information

### Comment

Schiff base complexes are one of the most important stereochemical models in transition metal coordination chemistry, with the ease of preparation and structural variations (Granovski et al., 1993). Metal derivatives of the Schiff bases have been studied extensively, and Ni(II) and Cu(II) complexes play a major role in both synthetic and structural research (Elmali et al., 2000; Blower et al., 1998).

The molecular structure of the title compound is shown in Fig. 1. The asymmetric unit comprises half of a Schiff base complex. The bond lengths (Allen et al., 1987) and angles are within the normal ranges. The geometry around the Cu^II^ ion is slightly distorted square-planar for which the coordination is a N_2_O_2_ donor set of the Schiff base ligand. The dihedral angle between the mean planes of the centeral aromatic ring with the two symmetry-related outer rings is 5.1 (2)°. The crystal structure is stabilized by intermolecular π–π interactions [Cg1···Cg3^i^ = 3.594 (2)Å, (i) -x, 1 - y, -z; Cg2···Cg2^i^ = 3.6431 (16)Å, Cg2···Cg3^i^ = 3.466 (2)Å, Cg1, Cg2, and Cg3 are the centroids of Cu1/N1/C8/C8A/N1A, C1–C6, and Cu1/O1/C1/C6/C7/N1, respectively.

### Experimental

The title compound was synthesized by adding bis(3-ethoxysalicylidene)-4,5- dimethyl phenylenediamine (2 mmol) to a solution of CuCl_2_.4H_2_O (2 mmol) in ethanol (30 ml). The mixture was refluxed with stirring for half an hour. The resultant solution was filtered. Dark-green single crystals of the title compound suitable for *X*-ray structure determination were recrystallized from ethanol by slow evaporation of the solvents at room temperature over several days.

### Refinement

All hydrogen atoms were positioned geometrically with C-H = 0.93-0.97 Å and included in a riding model approximation with U_iso_ (H) = 1.2 or 1.5 U_eq_ (C). A rotating group model was applied to the methyl groups.

### Crystal data

**Table:**

[Cu(C_26_H_26_N_2_O_4_)]	*F*(000) = 1028
*M**_r_* = 494.03	*D*_x_ = 1.294 Mg m^−^^3^
Monoclinic, *C*2/*c*	Mo *K*α radiation, λ = 0.71073 Å
Hall symbol: -C 2yc	Cell parameters from 2273 reflections
*a* = 14.9755 (7) Å	θ = 2.5–27.5°
*b* = 15.8803 (7) Å	µ = 0.89 mm^−^^1^
*c* = 12.2264 (6) Å	*T* = 296 K
β = 119.285 (2)°	Block, green
*V* = 2536.0 (2) Å^3^	0.27 × 0.21 × 0.11 mm
*Z* = 4	

### Data collection

**Table:**

Bruker SMART APEXII CCD area-detector diffractometer	3157 independent reflections
Radiation source: fine-focus sealed tube	1910 reflections with *I* > 2σ(*I*)
graphite	*R*_int_ = 0.049
φ and ω scans	θ_max_ = 28.3°, θ_min_ = 2.0°
Absorption correction: multi-scan (*SADABS*; Bruker, 2005)	*h* = −16→20
*T*_min_ = 0.982, *T*_max_ = 0.992	*k* = 0→21
3157 measured reflections	*l* = −16→0

### Refinement

**Table:**

Refinement on *F*^2^	Primary atom site location: structure-invariant direct methods
Least-squares matrix: full	Secondary atom site location: difference Fourier map
*R*[*F*^2^ > 2σ(*F*^2^)] = 0.066	Hydrogen site location: inferred from neighbouring sites
*wR*(*F*^2^) = 0.147	H-atom parameters constrained
*S* = 1.05	*w* = 1/[σ^2^(*F*_o_^2^) + (0.0549*P*)^2^ + 4.1197*P*] where *P* = (*F*_o_^2^ + 2*F*_c_^2^)/3
3157 reflections	(Δ/σ)_max_ = 0.001
152 parameters	Δρ_max_ = 0.43 e Å^−^^3^
0 restraints	Δρ_min_ = −0.46 e Å^−^^3^

### Special details

**Table:**

Geometry. All esds (except the esd in the dihedral angle between two l.s. planes) are estimated using the full covariance matrix. The cell esds are taken into account individually in the estimation of esds in distances, angles and torsion angles; correlations between esds in cell parameters are only used when they are defined by crystal symmetry. An approximate (isotropic) treatment of cell esds is used for estimating esds involving l.s. planes.
Refinement. Refinement of F^2^ against ALL reflections. The weighted R-factor wR and goodness of fit S are based on F^2^, conventional R-factors R are based on F, with F set to zero for negative F^2^. The threshold expression of F^2^ > 2sigma(F^2^) is used only for calculating R-factors(gt) etc. and is not relevant to the choice of reflections for refinement. R-factors based on F^2^ are statistically about twice as large as those based on F, and R- factors based on ALL data will be even larger.

### Fractional atomic coordinates and isotropic or equivalent isotropic displacement parameters (Å^2^)

**Table:**

	*x*	*y*	*z*	*U*_iso_*/*U*_eq_	
Cu1	0.0000	0.51186 (3)	0.2500	0.0401 (2)	
O1	0.06365 (19)	0.42620 (14)	0.2039 (2)	0.0464 (6)	
N1	0.0519 (2)	0.60241 (16)	0.1905 (3)	0.0401 (7)	
O2	0.1286 (2)	0.28916 (14)	0.1524 (3)	0.0591 (8)	
C1	0.1134 (3)	0.4353 (2)	0.1421 (3)	0.0395 (8)	
C2	0.1501 (3)	0.3619 (2)	0.1111 (3)	0.0444 (9)	
C3	0.2022 (3)	0.3667 (3)	0.0445 (4)	0.0548 (10)	
H3A	0.2271	0.3177	0.0272	0.066*	
C4	0.2178 (3)	0.4435 (3)	0.0029 (4)	0.0590 (11)	
H4A	0.2514	0.4457	−0.0440	0.071*	
C5	0.1843 (3)	0.5156 (3)	0.0305 (4)	0.0531 (10)	
H5A	0.1958	0.5670	0.0028	0.064*	
C6	0.1315 (3)	0.5138 (2)	0.1013 (3)	0.0411 (8)	
C7	0.0998 (3)	0.5921 (2)	0.1269 (3)	0.0449 (9)	
H7A	0.1145	0.6403	0.0954	0.054*	
C8	0.0270 (3)	0.6835 (2)	0.2165 (3)	0.0436 (9)	
C9	0.0525 (3)	0.7599 (2)	0.1837 (4)	0.0544 (10)	
H9A	0.0881	0.7601	0.1392	0.065*	
C10	0.0264 (3)	0.8356 (2)	0.2158 (4)	0.0621 (13)	
C11	0.0545 (4)	0.9164 (3)	0.1760 (5)	0.0899 (18)	
H11A	0.0925	0.9516	0.2481	0.135*	
H11B	−0.0067	0.9451	0.1169	0.135*	
H11C	0.0957	0.9041	0.1376	0.135*	
C12	0.1611 (3)	0.2112 (2)	0.1270 (4)	0.0537 (10)	
H12A	0.2352	0.2095	0.1671	0.064*	
H12B	0.1336	0.2044	0.0373	0.064*	
C13	0.1234 (4)	0.1426 (3)	0.1764 (5)	0.0774 (14)	
H13A	0.1490	0.0896	0.1659	0.116*	
H13B	0.0499	0.1419	0.1313	0.116*	
H13C	0.1469	0.1521	0.2638	0.116*	

### Atomic displacement parameters (Å^2^)

**Table:**

	*U*^11^	*U*^22^	*U*^33^	*U*^12^	*U*^13^	*U*^23^
Cu1	0.0526 (4)	0.0263 (3)	0.0492 (4)	0.000	0.0309 (3)	0.000
O1	0.0652 (17)	0.0318 (12)	0.0572 (16)	0.0001 (11)	0.0415 (15)	0.0021 (11)
N1	0.0440 (18)	0.0284 (15)	0.0416 (17)	0.0005 (12)	0.0161 (15)	0.0026 (12)
O2	0.084 (2)	0.0334 (14)	0.084 (2)	0.0090 (13)	0.0601 (18)	0.0010 (13)
C1	0.041 (2)	0.0384 (19)	0.042 (2)	−0.0003 (15)	0.0224 (18)	0.0007 (15)
C2	0.050 (2)	0.045 (2)	0.044 (2)	0.0025 (17)	0.0278 (19)	−0.0011 (16)
C3	0.056 (3)	0.057 (2)	0.061 (3)	0.002 (2)	0.037 (2)	−0.006 (2)
C4	0.058 (3)	0.071 (3)	0.066 (3)	−0.005 (2)	0.045 (2)	−0.002 (2)
C5	0.057 (2)	0.053 (2)	0.052 (2)	−0.0105 (19)	0.029 (2)	0.0052 (19)
C6	0.0423 (19)	0.0403 (18)	0.0402 (19)	−0.0024 (16)	0.0199 (16)	0.0006 (16)
C7	0.049 (2)	0.040 (2)	0.042 (2)	−0.0098 (16)	0.0197 (19)	0.0053 (16)
C8	0.048 (2)	0.0262 (17)	0.042 (2)	−0.0007 (15)	0.0111 (17)	0.0014 (15)
C9	0.060 (3)	0.0328 (19)	0.054 (2)	−0.0060 (18)	0.015 (2)	0.0081 (17)
C10	0.069 (3)	0.0271 (19)	0.053 (3)	−0.0036 (18)	0.001 (2)	0.0032 (16)
C11	0.116 (4)	0.032 (2)	0.083 (4)	−0.015 (2)	0.019 (3)	0.010 (2)
C12	0.055 (2)	0.044 (2)	0.064 (3)	0.0131 (18)	0.031 (2)	−0.0042 (18)
C13	0.101 (4)	0.039 (2)	0.103 (4)	0.011 (2)	0.058 (3)	0.000 (2)

### Geometric parameters (Å, °)

**Table:**

Cu1—O1^i^	1.898 (2)	C6—C7	1.419 (5)
Cu1—O1	1.898 (2)	C7—H7A	0.9300
Cu1—N1^i^	1.938 (3)	C8—C9	1.389 (5)
Cu1—N1	1.938 (3)	C8—C8^i^	1.406 (7)
O1—C1	1.303 (4)	C9—C10	1.379 (5)
N1—C7	1.301 (5)	C9—H9A	0.9300
N1—C8	1.419 (4)	C10—C10^i^	1.404 (9)
O2—C2	1.361 (4)	C10—C11	1.504 (5)
O2—C12	1.419 (4)	C11—H11A	0.9600
C1—C2	1.417 (5)	C11—H11B	0.9600
C1—C6	1.417 (5)	C11—H11C	0.9600
C2—C3	1.378 (5)	C12—C13	1.484 (6)
C3—C4	1.385 (5)	C12—H12A	0.9700
C3—H3A	0.9300	C12—H12B	0.9700
C4—C5	1.358 (5)	C13—H13A	0.9600
C4—H4A	0.9300	C13—H13B	0.9600
C5—C6	1.430 (5)	C13—H13C	0.9600
C5—H5A	0.9300		
			
O1^i^—Cu1—O1	88.42 (14)	N1—C7—H7A	117.2
O1^i^—Cu1—N1^i^	93.93 (11)	C6—C7—H7A	117.2
O1—Cu1—N1^i^	174.47 (11)	C9—C8—C8^i^	119.1 (2)
O1^i^—Cu1—N1	174.47 (11)	C9—C8—N1	126.1 (4)
O1—Cu1—N1	93.93 (11)	C8^i^—C8—N1	114.82 (19)
N1^i^—Cu1—N1	84.17 (17)	C10—C9—C8	121.6 (4)
C1—O1—Cu1	127.2 (2)	C10—C9—H9A	119.2
C7—N1—C8	122.0 (3)	C8—C9—H9A	119.2
C7—N1—Cu1	124.8 (2)	C9—C10—C10^i^	119.4 (3)
C8—N1—Cu1	113.1 (2)	C9—C10—C11	119.2 (4)
C2—O2—C12	119.4 (3)	C10^i^—C10—C11	121.4 (3)
O1—C1—C2	118.0 (3)	C10—C11—H11A	109.5
O1—C1—C6	124.3 (3)	C10—C11—H11B	109.5
C2—C1—C6	117.6 (3)	H11A—C11—H11B	109.5
O2—C2—C3	124.8 (3)	C10—C11—H11C	109.5
O2—C2—C1	114.0 (3)	H11A—C11—H11C	109.5
C3—C2—C1	121.2 (3)	H11B—C11—H11C	109.5
C2—C3—C4	120.7 (4)	O2—C12—C13	108.2 (3)
C2—C3—H3A	119.6	O2—C12—H12A	110.1
C4—C3—H3A	119.6	C13—C12—H12A	110.1
C5—C4—C3	120.2 (4)	O2—C12—H12B	110.1
C5—C4—H4A	119.9	C13—C12—H12B	110.1
C3—C4—H4A	119.9	H12A—C12—H12B	108.4
C4—C5—C6	121.0 (4)	C12—C13—H13A	109.5
C4—C5—H5A	119.5	C12—C13—H13B	109.5
C6—C5—H5A	119.5	H13A—C13—H13B	109.5
C1—C6—C7	123.4 (3)	C12—C13—H13C	109.5
C1—C6—C5	119.2 (3)	H13A—C13—H13C	109.5
C7—C6—C5	117.3 (3)	H13B—C13—H13C	109.5
N1—C7—C6	125.7 (3)		
			
O1^i^—Cu1—O1—C1	169.0 (3)	C2—C1—C6—C7	−179.4 (3)
N1—Cu1—O1—C1	−6.0 (3)	O1—C1—C6—C5	−177.9 (3)
O1—Cu1—N1—C7	8.1 (3)	C2—C1—C6—C5	0.7 (5)
N1^i^—Cu1—N1—C7	−177.1 (4)	C4—C5—C6—C1	−0.6 (6)
O1—Cu1—N1—C8	−175.5 (2)	C4—C5—C6—C7	179.5 (3)
N1^i^—Cu1—N1—C8	−0.69 (17)	C8—N1—C7—C6	177.1 (3)
Cu1—O1—C1—C2	−176.4 (2)	Cu1—N1—C7—C6	−6.9 (5)
Cu1—O1—C1—C6	2.2 (5)	C1—C6—C7—N1	0.7 (6)
C12—O2—C2—C3	0.1 (6)	C5—C6—C7—N1	−179.4 (3)
C12—O2—C2—C1	179.8 (3)	C7—N1—C8—C9	−2.6 (6)
O1—C1—C2—O2	−0.6 (5)	Cu1—N1—C8—C9	−179.1 (3)
C6—C1—C2—O2	−179.3 (3)	C7—N1—C8—C8^i^	178.5 (4)
O1—C1—C2—C3	179.1 (3)	Cu1—N1—C8—C8^i^	2.0 (5)
C6—C1—C2—C3	0.5 (5)	C8^i^—C8—C9—C10	0.2 (6)
O2—C2—C3—C4	178.0 (4)	N1—C8—C9—C10	−178.7 (3)
C1—C2—C3—C4	−1.7 (6)	C8—C9—C10—C10^i^	0.7 (7)
C2—C3—C4—C5	1.8 (7)	C8—C9—C10—C11	−179.0 (4)
C3—C4—C5—C6	−0.6 (6)	C2—O2—C12—C13	−177.2 (4)
O1—C1—C6—C7	2.0 (6)		

Symmetry codes: (i) −*x*, *y*, −*z*+1/2.
